# Long term outcome of high-risk neuroblastoma patients after immunotherapy with antibody ch14.18 or oral metronomic chemotherapy

**DOI:** 10.1186/1471-2407-11-21

**Published:** 2011-01-18

**Authors:** Thorsten Simon, Barbara Hero, Andreas Faldum, Rupert Handgretinger, Martin Schrappe, Thomas Klingebiel, Frank Berthold

**Affiliations:** 1 Children's Hospital, University of Cologne, Cologne, Germany; 2 Institute for Medical Biostatistics and Clinical Research, University of Münster, Münster, Germany; 3 Children's Hospital, University of Tübingen, Tübingen, Germany; 4 Children's Hospital, University Medical Center Schleswig-Holstein, Campus Kiel, Kiel, Germany; 5 Johann Wolfgang Goethe University, Department of Pediatrics III, Frankfurt/Main, Germany

## Abstract

**Background:**

The treatment of high-risk neuroblastoma patients consists of multimodal induction therapy to achieve remission followed by consolidation therapy to prevent relapses. However, the type of consolidation therapy is still discussed controversial. We applied metronomic chemotherapy in the prospective NB90 trial and monoclonal anti-GD2-antibody (MAB) ch14.18 in the NB97 trial. Here, we present the long term outcome data of the patient cohort.

**Methods:**

A total of 334 stage 4 neuroblastoma patients one year or older were included. All patients successfully completed the induction therapy. In the NB90 trial, 99 patients received at least one cycle of the oral maintenance chemotherapy (NB90 MT, 12 alternating cycles of oral melphalan/etoposide and vincristine/cyclophosphamide). In the NB97 trial, 166 patients commenced the MAB ch14.18 consolidation therapy (six cycles over 12 months). Patients who received no maintenance therapy according to the NB90 protocol or by refusal in NB97 (n = 69) served as controls.

**Results:**

The median observation time was 11.11 years. The nine-year event-free survival rates were 41 ± 4%, 31 ± 5%, and 32 ± 6% for MAB ch14.18, NB90 MT, and no consolidation, respectively (p = 0.098). In contrast to earlier reports, MAB ch14.18 treatment improved the long-term outcome compared to no additional therapy (p = 0.038). The overall survival was better in the MAB ch14.18-treated group (9-y-OS 46 ± 4%) compared to NB90 MT (34 ± 5%, p = 0.026) and to no consolidation (35 ± 6%, p = 0.019). Multivariable Cox regression analysis revealed ch14.18 consolidation to improve outcome compared to no consolidation, however, no difference between NB90 MT and MAB ch14.18-treated patients was found.

**Conclusions:**

Follow-up analysis of the patient cohort indicated that immunotherapy with MAB ch14.18 may prevent late relapses. Finally, metronomic oral maintenance chemotherapy also appeared effective.

## Background

The prognosis of high-risk neuroblastoma patients has improved over the last decades. However, even after high intensive treatment only a few patients become long-term survivors [1-3]. Most high-risk patients develop relapse after initial response to induction treatment. Prevention of these relapses by additional conventional chemotherapy is limited due to cumulative toxicity. Thus, additional treatments to chemotherapy, surgery, and radiotherapy have to be sought. Metronomic low dose chemotherapy was considered to have the potential to prevent relapses with acceptable low toxicity. Therefore, an oral chemotherapy with cyclophosphamide, etoposide and melphalan was introduced in trial NB90. Monoclonal antibodies (MAB) directed against GD2 have offered another promising avenue of treatment [4-10]. Therefore, the chimeric human/mouse antibody ch14.18 was applied as consolidation treatment in pilot patients of the trial NB90 and all high-risk patients in the NB97. Early analysis of MAB ch14.18 consolidation in high-risk neuroblastoma patients did not demonstrate reduction of the recurrence rate [11,12]. Here, we present the long-term outcome of the cohort.

## Methods

A total of 334 patients of the Cooperative German Neuroblastoma Trials NB90 and NB97 were included in this analysis when they met the following inclusion criteria: (1) stage 4 neuroblastoma diagnosed according to the INSS criteria [13], (2) age at diagnosis one year or older, (3) diagnosis between September 01, 1989 and January 01, 2002, (4) treatment according to the NB90/NB97 neuroblastoma trials, (5) no event (relapse, progression, death, secondary malignant disease) during induction chemotherapy, (6) no combination of NB90 maintenance treatment and ch14.18 antibody, (7) no additional treatment with 13 cis-retinoic acid, and (8) informed parents' consent for treatment and the collection of data.

**Figure 1:**
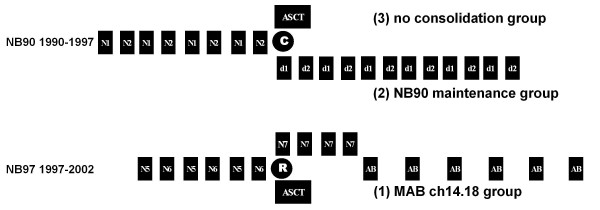
**Treatment scheme (for details of N1, N2, N5, N6, N7, D1, and D2 see 'Methods' text, C = choice, R = randomization, ASCT = myeloablative chemotherapy with autologous stem cell transplant, AB = antibody ch14.18)**.

NB90 induction chemotherapy consisted of four N1 chemotherapy cycles (cisplatin, etoposide, vindesine) and four N2 cycles (vincristine, dacarbacine, ifosfamide, doxorubicine) [1]. Myeloablative chemotherapy with autologuous stem cell transplantation (ASCT) was an option for patients in complete or very good partial remission. Patients not treated with ASCT received maintenance therapy consisting of alternating cycles D1 (oral melphalan 8 mg/m²/d days 1-5 and oral etoposide 100 mg/m²/d days 1-5) and D2 (intravenous vincristine 1.5 mg/m² day 1 and oral cyclophosphamide 150 mg/m²/d days 1-7) each month for one year [1]. In NB97, the NB90 induction chemotherapy was detoxified by reduction of the etoposide dose by 20 %, the doxorubicine infusion time from 48 to 4 hours on two consecutive days, and the total number of chemotherapy cycles from 8 to 6. Induction was followed by randomization either for myeloablative chemotherapy with stem cell transplantation (melphalan, etoposide, carboplatin) or four cycles of oral cyclophosphamide [14] (Figure 1). Radiotherapy was administered for bone metastases and non-progressing residual primary tumours in NB90. In the NB97 trial, radiotherapy was reserved for patients with residual MIBG-positive primary tumours only [15].

After initial treatment, all NB97 trial patients and a limited number of NB90 ASCT pilot patients were scheduled for treatment with the monoclonal anti-GD2-antibody ch14.18. This MAB was produced by BioInvent International AB (Lund, Sweden), vialled by the pharmaceutical company Rentschler (Langheim, Germany), and certified by the Paul-Ehrlich-Institute (Langen, Germany) for investigational use within a national trial. All hospitals collaborating in the German Neuroblastoma trials were qualified for antibody treatment when their local ethics committee had approved the antibody treatment. MAB ch14.18 was stored in two centres (Tubingen and Cologne) and was made available to the hospitals after induction chemotherapy documentation of the individual patient was complete. The MAB ch14.18 treatment regime consisted of an infusion of 20 mg/m²/d over 8-12 hours on five subsequent days. This cycle was repeated every 2 months for a total of six cycles. Concomitant intravenous morphine starting at a dose of 1.0 mg/kg/d was strongly recommended for pain control. In addition, other analgesic drugs (metamizol, paracetamol, tramadol) and cortisone could also be administered.

The "as treated" outcome analysis was based on the hypothesis that immunotherapy with MAB ch14.18 and oral maintenance chemotherapy in the NB90 trial share the concept of prolonged consolidation treatment of potential minimal residual disease. Therefore, we compared the survival data of the MAB ch14.18-treated group with the group receiving oral maintenance chemotherapy in the NB90 trial instead, as well as with those patients who received no further consolidation treatment after initial therapy. For this purpose, three groups were defined: (1) antibody ch14.18 group consisting of patients of trials NB90 and NB97 who received MAB-ch14.18-antibody treatment but no oral maintenance chemotherapy according to NB90; (2) oral NB90 maintenance chemotherapy group; (3) no consolidation therapy after induction chemotherapy and ASCT or induction chemotherapy only including all patients who neither received MAB ch14.18 nor oral maintenance chemotherapy according to NB90 trial.

Data were analyzed in May 2010 using the statistical package SPSS version 17.0.0. Note, that all statistical analyses are regarded as explorative, particularly all analyses of subgroups. Proportions were compared using the two-tailed chi² test or Fisher's exact test as appropriate. Means were compared using the Mann Whitney U-test. Survival curves were calculated according to Kaplan-Meier. Survival times between two or more groups were compared by the log-rank test. Event-free survival time was calculated as the time from diagnosis to event or last examination if the patient had no event. Relapse, progression, death, and secondary malignant disease were regarded as events. Overall survival time was calculated as the time from diagnosis to death or last examination if the patient survived. In the latter case, the survival time was assigned as censored. Multivariable Cox regression was applied to analyze the prognostic value of these risk factors with respect to event-free and overall survival. The following potential six explanatory prognostic factors were considered: (1) LDH at diagnosis (abnormal vs. normal as reference), (2) MYCN (amplified vs. not amplified as reference), (3) age at diagnosis (continuous), (4) protocol (NB90 vs. NB97 as reference), (5) treatment group (antibody ch14.18 group as reference vs. NB90 oral maintenance chemotherapy or no consolidation group), and (6) ASCT (yes vs. no as reference). Models were build using a stepwise variable selection procedure recommended by Collett [16]. In the first step, all parameters were tested one at a time in a univariate Cox regression. In the second step, all parameters that appeared to be important in step 1 were analyzed jointly by a Cox regression backward selection. In a third step, all parameters that were not important in step 1 were added, one at a time, to the parameters which were important in step 2. After the third step, the selection process due to Collett ended since no additional risk factors were found. A p-value of p≤ 0.05 in the score test served as the inclusion criterion and a p-value of p>0.10 in the likelihood ratio test served as the exclusion criterion.

## Results

### Patients' characteristics

A total of 334 patients were included in this follow-up study (see Table 1). The three treatment groups (ch14.18, oral NB90 maintenance, and no consolidation group) were not different in age, gender distribution, MYCN status, and status prior to consolidation therapy. By definition, ASCT was unbalanced between the groups: The frequency of patients who underwent ASCT prior to consolidation therapy was 62.0%, 0%, and 60.8% in the groups of ch14.18 treatment, oral NB90 maintenance chemotherapy, and no consolidation, respectively. The median observation time was 11.11 years (range: 2.27 - 18.57 years).

**Table 1:** Patients' characteristics of the three treatment groups analyzed in this follow-up study

		Treatment group			Total	p-value
		Antibody18 ch14.18	Oral NB90 maintenance18 chemotherapy	No consolidation		
No. of patients		166	99	69	334	
						
Age at diagnosis	median (years)	3.2	2.9	2.9	3.2	.262
	range (years)	1.0-20.6	1.0-15.0	1.0-11.2	1.0-20.6	
						
Sex	male	95	54	43	192	.602
	female	71	45	26	142	
						
Protocol	NB90	25	99	53	177	<.001
	NB97	141	0	16	157	
						
ASCT	no	63	99	27	189	<.001
	yes	103	0	42	145	
						
MYCN status	Normal	124	56	34	214	.358
	Amplified	38	15	16	69	
	Not known	4	28	19	51	
						
Disease status after initial treatment	CR/VGPR	134	81	52	267	.567
	PR	27	17	16	60	
	MR/SD	5	1	1	7	

### Consolidation treatment

A total of 164 patients received at least one antibody cycle. The two remaining patients of the ch14.18 group experienced relapse while waiting for the first cycle but were included in the group according to the intention-to-treat approach. Due to relapses, the number of patients who received the next antibody cycles was decreasing: A total of 148, 133, 107, 100, and 83 patients received a 2^nd^, 3^rd^, 4^th^, 5^th^, and 6^th ^ch14.18 cycle, respectively. One patient had more than 6 antibody cycles. MAB ch14.18 treatment was discontinued prematurely in 6 children because their parents felt the side-effects were unacceptable although the consulting physician recommended continuation of MAB ch14.18 treatment. Antibody treatment was stopped for medical reasons because of capillary leak syndrome in 2 patients and infectious hepatitis not related to antibody treatment in 1 patient. The median dose of MAB ch14.18 was 20 mg/m²/d (range: 12 to 40 mg/m²/d). Due to tolerance during preparation, 2 patients had < 18 mg/m²/d (< 90% of the scheduled dose). No patient had dose reduction due to side-effects. Two patients received >22 mg/m²xd (>110% of the scheduled dose). The median time interval between antibody cycles or the preceding chemotherapy to first antibody cycle was 65.5 days (range: 39.5 - 343 days; planned: 60 days). Detailed toxicity data have been reported earlier [11]. The presence of allergic symptoms such as rash, conjunctivitis, and pruritus during at least one MAB ch14.18 cycle had no clear impact on the outcome compared to no allergic symptoms (9-year EFS 44.2 ± 4.8% with symptoms of allergy vs. 35.0 ± 6.2%, p = 0.105 and 9-year OS 50.0 ± 4.9% with allergy vs. 38.3 ± 6.5%, p = 0.080).

### Outcome

**Figure 2:**
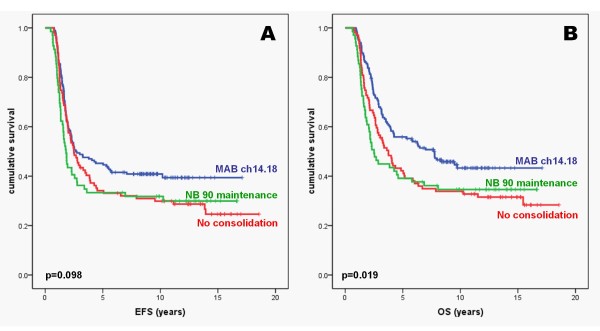
**Event-free survival (A) and overall survival (B) of 334 patients comparing ch14.18 consolidation (blue line), NB90 oral maintenance therapy (green line), and no consolidation (red line)**.

The global 5-year-EFS rate was 39.5 ± 2.7% and the 5-year-OS rate was 48.4 ± 2.7%. Late events were rare resulting in a very similar 9-year-EFS rate of 36.1 ± 2.6% and a 9-year-OS rate of 40.1 ± 2.7%. Results of univariate analysis comparing the three treatment groups are found in Table 2. In the entire cohort, paired log-rank test demonstrated a lower event rate after ch14.18 consolidation compared to no consolidation (p = 0.038) but no clear difference between MAB ch14.18 treatment and oral NB90 maintenance chemotherapy (p = 0.147, Figure 2a). The overall survival rate was better after antibody ch14.18 consolidation compared to no consolidation therapy (p = 0.015) and to oral NB90 maintenance chemotherapy (p = 0.023, Figure 2b). Extensive subgroup analysis echoed the results of the global analysis and demonstrated better overall survival after MAB ch14.18 consolidation in patients without MYCN amplification, patients in CR/VGPR after induction, and patients without residual bone marrow involvement after induction. Moreover, 78 patients of trial NB97 who underwent MAB ch14.18 consolidation after ASCT also had a better OS rate compared to 99 patients who underwent NB90 maintenance chemotherapy (p = 0.035). Accordingly, multivariable analysis found better EFS and OS of MAB ch14.18 consolidation compared to no consolidation therapy. In contrast, no outcome difference was detected between MAB ch14.18 consolidation and 12 months of oral chemotherapy according to NB90 (Table 3). The well-known risk factors, namely high LDH at diagnosis, MYCN amplification, and higher age at diagnosis, were independently associated with poor outcome; whereas, ASCT was found to be the only factor to be associated with better outcome.

**Table 2:** Results of global and subgroup analysis

Subgroup defined by	Patient number		Antibody ch14.18 group	Maintenance chemotherapy group	No consolidation group	Log-rank p-value
All patients	344	5yEFS	45.2 ± 3.9%	34.1 ± 4.8%	33.3 ± 5.7%	.098
		5yOS	55.8 ± 3.9%	42.2 ± 5.0%	39.1 ± 5.9%	.019
		9yEFS	40.9 ± 3.8%	31.0 ± 4.7%	31.8 ± 5.6%	.098
		9yOS	45.8 ± 4.0%	33.9 ± 4.8%	34.6 ± 5.7%	.019
						
With ASCT	145	5yEFS	50.5 ± 4.9%		38.1 ± 7.5%	.241
		5yOS	58.3 ± 4.9%		45.2 ± 7.7%	.152
		9yEFS	44.5 ± 4.9%		38.1 ± 7.5%	.241
		9yOS	47.0 ± 5.0%		40.5 ± 7.6%	.152
						
No ASCT	189	5yEFS	36.5 ± 6.1%	34.1 ± 4.8%	25.9 ± 8.4%	.133
		5yOS	51.9 ± 6.3%	42.2 ± 5.0%	29.6 ± 8.8%	.094
		9yEFS	34.9 ± 6.0%	31.0 ± 4.7%	21.6 ± 8.1%	.133
		9yOS	43.9 ± 6.5%	33.9 ± 4.8%	25.4 ± 8.5%	.094
						
NB90	177	5yEFS	48.0 ± 10%	34.1 ± 4.8%	37.7 ± 6.7%	.300
		5yOS	52.0 ± 10%	42.2 ± 5.0%	41.5 ± 6.8%	.250
		9yEFS	44.0 ± 9.9%	31.0 ± 4.7%	33.6 ± 6.5%	.300
		9yOS	48.0 ± 10.0%	33.9 ± 4.8%	37.6 ± 6.7%	.250
						
NB97	157	5yEFS	44.7 ± 4.2%		18.8 ± 9.8%	.067
		5yOS	56.5 ± 4.2%		31.3 ± 11.6%	.050
		9yEFS	40.3 ± 4.1%		18.8 ± 9.8%	.067
		9yOS	45.3 ± 4.3%		23.4 ± 11.0%	.050
						
NB90 maintenance and NB97 ASCT+ch14.18 patients	177	5yEFS	51.3 ± 5.7%	34.1 ± 4.8%		.106
		5yOS	60.3 ± 5.5%	42.2 ± 5.0%		.035
		9yEFS	44.7 ± 5.6%	31.0 ± 4.7%		.106
		9yOS	46.6 ± 5.7%	33.9 ± 4.8%		.035
						
MYCN not amplified	214	5yEFS	48.4 ± 4.5%	37.0 ± 6.5%	35.3 ± 8.2%	.166
		5yOS	61.9 ± 4.4%	47.8 ± 6.5%	44.1 ± 8.5%	.041
		9yEFS	45.2 ± 4.5%	31.4 ± 6.3%	35.3 ± 8.2%	.166
		9yOS	52.0 ± 4.6%	34.9 ± 6.4%	38.0 ± 8.4%	.041
						
MYCN amplified	69	5yEFS	34.2 ± 7.7%	20.0 ± 10.3%	31.3 ± 11.6%	.997
		5yOS	36.8 ± 7.8%	26.7 ± 11.4%	31.3 ± 11.6%	.902
		9yEFS	25.5 ± 7.2%	20.0 ± 10.3%	31.3 ± 11.6%	.997
		9yOS	24.9 ± 7.2%	20.0 ± 10.3%	31.3 ± 11.6%	.902
						
CR/VGPR after induction	267	5yEFS	47.0 ± 4.3%	34.4 ± 5.3%	36.5 ± 6.7%	.185
		5yOS	58.2 ± 4.3%	37.9 ± 5.4%	42.3 ± 6.9%	.032
		9yEFS	41.7 ± 4.3%	30.5 ± 5.2%	34.5 ± 6.6%	.185
		9yOS	45.8 ± 4.4%	32.8 ± 5.3%	36.3 ± 6.7%	.032
						
CR after induction	166	5yEFS	54.9 ± 5.5 %	40.5 ± 7.1 %	48.6 ± 8.4 %	.392
		5yOS	62.2 ± 5.4 %	46.4 ± 7.2 %	57.1 ± 8.4 %	.218
		9yEFS	49.8 ± 5.5%	36.1 ± 6.9%	45.7 ± 8.4%	.392
		9yOS	51.6 ± 5.6%	37.8 ± 7.0%	48.6 ± 8.4%	.218
						
PR after induction	60	5yEFS	37.0 ± 9.3%	29.4 ± 11.1%	25.0 ± 10.8%	.644
		5yOS	46.9 ± 9.8%	58.8 ± 11.9%	31.3 ± 11.6%	.632
		9yEFS	37.0 ± 9.3%	29.4 ± 11.1%	25.0 ± 10.8%	.644
		9yOS	46.9 ± 9.8%	35.3 ± 11.6%	31.3 ± 11.6%	.632
						
No residual bone marrow involvement after induction	316	5yEFS	46.8 ± 4.0 %	36.0 ± 5.0 %	33.8 ± 5.7 %	.122
		5yOS	57.0 ± 4.0 %	43.4 ± 5.1 %	39.7 ± 5.9 %	.033
		9yEFS	42.1 ± 4.0%	32.6 ± 4.9%	32.2 ± 5.6%	.122
		9yOS	46.8 ± 4.1%	35.7 ± 5.0%	35.1 ± 5.8%	.033

**Table 3:** Results of multivariable Cox regression analysis

Factor	Number of patients included in the relevant step	p-value of the likelihood ratio test	hazard ratio	95% confidence interval of hazard ratio
Event free survival				
LDH at diagnosis (abnormal vs. normal)	325	.020	2.155	1.129 - 4.113
MYCN (amplified vs. normal)	264	.009	1.565	1.117 - 2.191
Age at diagnosis (continuous)	334	<.001	1.099	1.054 - 1.146
ASCT (yes vs. no)	334	.016	.647	.453 - .923
Consolidation treatment	334	.068		
NB90 maintenance vs. MAB ch14.18		.688	1.083	.733 - 1.601
No consolidation vs. MAB ch14.18		.021	1.625	1.077 - 2.453
Protocol (NB90 vs. NB97)	334	.106		


Overall survival				
LDH at diagnosis (abnormal vs. normal)	325	.016	2.315	1.170 - 4.578
MYCN (amplified vs. normal)	264	<.001	1.182	1.085 - 1.289
Age at diagnosis (continuous)	334	<.001	1.111	1.062 - 1.161
ASCT (yes vs. no)	334	.036	.673	.464 - .975
Consolidation treatment	334	.031		
NB90 maintenance vs. MAB ch14.18		.182	1.317	.879 - 1.975
No consolidation vs. MAB ch14.18		.011	1.737	1.134 - 2.661
Protocol (NB90 vs. NB97)	334	.162		

## Discussion

This follow-up analysis of the German neuroblastoma trials demonstrated a possible benefit of antibody ch14.18-based consolidation therapy compared to no consolidation therapy on event-free and overall survival. Of note is that a difference in event-free survival was not found in a previous analysis performed in 2004 [11]. The possible explanation is that antibody therapy can prevent late relapses in patients with minimal residual disease.

Small patient series of consolidation therapy with single agent chimeric MAB ch14.18 [7,8,10] or murine MAB 3F8 [9] had shown encouraging results in high-risk neuroblastoma patients. Combinations of antibodies with cytokines [17,18], retinoic acid [19], or both [20]were tested and found to be tolerable. The randomized COG trial ANBL0032 was stopped after interim analysis because the antibody-containing arm was more effective than the retinoic acid standard arm [21]: Stage 4 neuroblastoma patients one year or older who received the immunotherapy combination had a better outcome (2-year EFS rate from randomization 63 ± 6%, 2-year OS rate 84 ± 4%) compared to the standard arm with retinoic acid (2-year EFS rate 42 ± 6%, p = 0.0155; 2-year OS rate 76 ± 5%, p = 0.1006). In order to compare the results of our study to the ANBL0032 survival rates, we have recalculated the survival times of our patients from start of consolidation therapy. Patients of the ch14.18 group had a 2-year EFS rate from the first ch14.18 cycle of 50.0 ± 3.9% and a 2-year-OS rate of 70.1 ± 3.6%. The NB90 maintenance group achieved a 2-year EFS from first continuation chemotherapy cycle of 46.5 ± 5.0% (p = 0.218) and a 2-year OS rate of 58.6 ± 5.0% (p = 0.028). Many factors may explain the lower survival rates in our study: (1) randomized design of ANBL0032 vs. nonrandomized retrospective analysis in NB90/97; (2) comparison of ch14.18-containing therapy to retinoic acid in the ANBL0032 trial vs. metronomic oral chemotherapy in NB90/97, and (3) combination of ch14.18, retinoic acid, IL2, and GM-CSF vs. ch14.18 alone in NB90/NB97.

Further, data on anti-mouse antibodies are not available for both trials. One might expect that patients with allergic reactions develop neutralizing antibodies resulting in inferior outcome. However, our data do not confirm such an effect. We found a trend for better outcome of patients who developed allergic symptoms.

Our follow-up analysis has limitations because of the retrospective nonrandomized design. However, patients were treated in three different well defined groups and free choice of continuation therapy was not possible. Except ASCT, all other major risk factors were well balanced between the three treatment groups (Table 1). The strength of this analysis is that MAB ch14.18 has been used as a single agent. Thus, the question arises as to exactly what MAB ch14.18 contributed to the beneficial effect of the immunotherapy combination in the ANBL0032 trial.

Of note is that MAB ch14.18 was as effective as metronomic 12 months oral maintenance chemotherapy of the NB90 trial despite the fact that more than half of the patients in the ch14.18 group, but none in the NB90 maintenance group, had undergone ASCT. It has been shown in randomized trials that ASCT improves the outcome of high risk neuroblastoma patients [3,14]. The multivariate analysis confirmed an independent impact of both consolidation therapy and ASCT. Therefore, one would actually expect an inferior outcome for NB90 maintenance patients without ASCT compared to the ch14.18 group including 61% ASCT patients which is not the case.

## Conclusions

Our data clearly demonstrate that no consolidation therapy is associated with worse outcome in high-risk neuroblastoma patients. Considering the result of this and earlier [11] analysis, single agent ch14.18 consolidation appeared to prevent late relapses. Today, the most effective way of antibody based maintenance therapy seems to be a combination immunotherapy with MAB ch14.18, cytokines, and retinoic acid [21]. But these results need confirmation by at least another randomized trial. Further, metronomic low dose oral chemotherapy consolidation was found as effective as MAB ch14.18 consolidation in this retrospective analysis and, therefore, also warrants further evaluation. Prospective clinical trials must demonstrate if the concept of low dose metronomic chemotherapy is feasible and effective after ASCT and in combination with immunotherapy.

## Competing interests

The authors declare that they have no competing interests.

## Authors' contributions

TS collected and analyzed the data, and wrote the manuscript as corresponding author. BH contributed to the preparation of the trial, collection and analysis of the data, and writing of the manuscript. AF is the trial statistician and strongly contributed to the analysis of the data and preparation of the manuscript. RH and MS were strongly involved in the designing and conduction of the trial. They also contributed to the preparation of the manuscript. FB is the principal investigator of both national neuroblastoma trial, contributed to preparation and conduction of the trials as well as the preparation of the manuscript. All authors read and approved the final manuscript.

## Pre-publication history

The pre-publication history for this paper can be accessed here:

http://www.biomedcentral.com/1471-2407/11/21/prepub
